# Supplementary material for: Content-rich biological network constructed by mining PubMed abstracts
Source: BMC Bioinformatics. 2004 Oct 8;5:147. doi: 10.1186/1471-2105-5-147 (PMC528731; doi:10.1186/1471-2105-5-147)
Supplement: Additional File 2 — The original results of the above study (non-essential files are deleted to keep the file size under the limit set by BMC bioinformatics). [file 1471-2105-5-147-S2.bz2 › chilibotAdditionalFile2/dip20/149ID9171381E537/html/VIRB7_VIRB9.html]

 


 **VIRB7** and **VIRB9** 
  
Found 20 abstracts in PubMed, retrieved 20.  
 

 What does Google say? 
 PDF only 
| .edu only 

---

**Interactive relationship** (e.g. stimulation, inhibition, etc)

**Neutral relationship**- Delineation of the interaction domains of Agrobacterium tumefaciens  **VirB7**  and  **VirB9**  by use of the yeast two hybrid assay.  Ref: 9171381 J Bacteriol, 1997
- The interaction between  **VirB9**  and  **VirB7**  also was observed in the yeast two hybrid system.  Ref: 9023204 J Bacteriol, 1997
- Our results are consistent with a model in which  **VirB7**  stabilizes  **VirB9**  by formation of a covalent intermolecular cross link.  Ref: 8655495 J Bacteriol, 1996
- The lipoprotein  **VirB7**  interacts with  **VirB9**  in the membranes of Agrobacterium tumefaciens.  Ref: 9023204 J Bacteriol, 1997
- Immunoprecipitation with  **VirB9**  specific antiserum isolated the heterodimeric  **VirB9**   **VirB7**  complex.  Ref: 9023204 J Bacteriol, 1997
- We now demonstrate that  **VirB7**  and  **VirB9**  interact in yeast, and this interaction does not require the cysteine residues essential for the disulfide linkage.  Ref: 9171381 J Bacteriol, 1997
- By using defined segments in fusion constructions, we mapped the  **VirB7**  interaction domain of  **VirB9**  to residues 173 to 275.  Ref: 9171381 J Bacteriol, 1997
- Membrane attachment of  **VirB9**   **VirB7**  may be conferred by lipoprotein modification, since labeling with 3H palmitic acid in A. tumefaciens verified that  **VirB7**  is a lipoprotein associated with  **VirB9** .  Ref: 9023204 J Bacteriol, 1997
- We established interactions between VirB11 an inner membrane pore forming protein,  **VirB9**  a periplasmic protein, and  **VirB7**  an outer membrane associated lipoprotein and putative pilus component.  Ref: 12177441 Proc Natl Acad Sci U S A, 2002
- Two types of dissociable complexes were visualized i a 36 kDa complex corresponding in size to a  **VirB7**   **VirB9**  heterodimer and ii an 84 kDa complex corresponding in size to a  **VirB7**   **VirB9**  PhoA293 heterodimer.  Ref: 8755505 Proc Natl Acad Sci U S A, 1996
- We recently demonstrated that  **VirB7**  and  **VirB9**  form a protein complex linked by a disulfide bond between cysteine 24 of  **VirB7**  and cysteine 262 of  **VirB9**  L.  Ref: 9171381 J Bacteriol, 1997
- Two dimensional gel electrophoresis identified one complex as a heterodimer of  **VirB9**  and  **VirB7**  covalently linked by a disulfide bond, as well as  **VirB7**  homodimers and monomers.  Ref: 9023204 J Bacteriol, 1997
- **VirB7**  assembles as a disulfide cross linked homodimer that associates with the T pilus and a  **VirB7**   **VirB9**  heterodimer that stabilizes other VirB proteins during biogenesis of the secretion machine.  Ref: 12700266 J Bacteriol, 2003
- Extracts of AS induced cells electrophoresed under nonreducing conditions possessed undetectable levels of the 32 kDa  **VirB9**  and 4.5 kDa  **VirB7**  monomers and instead possessed a 36 kDa complex that cross reacted with both  **VirB7**  and  **VirB9**  antisera and accumulated as a function of  **virB7**  expression.  Ref: 8655495 J Bacteriol, 1996
- First, a precise deletion of  **virB7**  from the pTiA6NC plasmid of wild type strain A348 was correlated with significant reductions in the steady state levels of several VirB proteins, including VirB4,  **VirB9** , VirB10, and VirB11.  Ref: 8655495 J Bacteriol, 1996

**Non-interactive relationship** (e.g. studied together, co-existance, homology, etc.)

- Using strains that contain a deletion in a defined virB gene and strains that express specific VirB proteins, we demonstrate that the 36 kDa band is composed of  **VirB9**  and  **VirB7**  that are linked to each other by a disulfide bond.  Ref: 8799123 Proc Natl Acad Sci U S A, 1996
- ii a delta virB operon mutant expressing  **virB7**  and virB8 from Plac and  **virB9** , virB10, and virB11 from PvirB.  Ref: 8655495 J Bacteriol, 1996
- The second class contains putative translocation complex core components VirB8,  **VirB9** , and VirB10 in the high molecular mass portion of the gel larger than 232 kDa, as well as  **VirB7** .  Ref: 12177443 Proc Natl Acad Sci U S A, 2002
- Incubation with reducing agent split the complex into its constituent  **VirB9**  and  **VirB7** , which further confirmed linkage via cysteine residues.  Ref: 9023204 J Bacteriol, 1997
- To examine the biological importance of VirB6 complex formation for type IV secretion, we monitored the effects of nonstoichiometric VirB6 production and the synthesis of VirB6 derivatives with 4 residue insertions VirB6.i4 on  **VirB7**  and  **VirB9**  multimerization, T pilus assembly, and substrate transfer.  Ref: 12700266 J Bacteriol, 2003
- Multiple insertions throughout VirB1, VirB5,  **VirB7** ,  **VirB9**  and VirB10 indicated that these proteins primarily face the periplasm.  Ref: 9484895 Mol Microbiol, 1998
- We further report that  **VirB7**  dependent stabilization of  **VirB9**  is correlated with the ability of these two proteins to dimerize via formation of a disulfide bridge between reactive Cys 24 and Cys 262 residues, respectively.  Ref: 8755505 Proc Natl Acad Sci U S A, 1996
- Agrobacterium tumefaciens  **VirB7**  and  **VirB9**  form a disulfide linked protein complex.  Ref: 8799123 Proc Natl Acad Sci U S A, 1996
- However, cross linking and coimmunoprecipitation showed a close association of cell bound VirB1\* with the  **VirB9**   **VirB7**  heterodimer, a membrane associated component of the T complex transfer machinery.  Ref: 9023203 J Bacteriol, 1997
- **VirB9**  and  **VirB7**  showed equal distribution between inner and outer membranes, in accord with their proposed association with the transmembrane VirB complex.  Ref: 9023204 J Bacteriol, 1997
- These results suggest that the outer membrane  **VirB7**   **VirB9**  complex interacts with the inner membrane proteins VirB8 and VirB10 for the assembly of the transport pore.  Ref: 10633111 J Bacteriol, 2000
- We studied interaction among four putative transport pore proteins,  **VirB7** , VirB8,  **VirB9**  and VirB10.  Ref: 10633111 J Bacteriol, 2000
- Whereas class I proteins VirB2,  **VirB7** ,  **VirB9** , and VirB10 were readily detected at 28 degrees C, class II proteins VirB1, VirB4, VirB5, VirB6, VirB8, VirB11, VirD2, and VirE2 were only detected after cell growth below 26 degrees C.  Ref: 11698374 J Bacteriol, 2001
- To test whether this method can be used to study interactions among the VirB membrane proteins, we studied the interaction of  **VirB7**  and  **VirB9**  in yeast.  Ref: 9171381 J Bacteriol, 1997
- Intermolecular disulfide bonds stabilize  **VirB7**  homodimers and  **VirB7**   **VirB9**  heterodimers during biogenesis of the Agrobacterium tumefaciens T complex transport apparatus.  Ref: 8755505 Proc Natl Acad Sci U S A, 1996
- Fractionation of  **VirB7**  containing complexes  **VirB7**   **VirB7**  homodimers and  **VirB7**   **VirB9**  heterodimers suggested that they may link the T pilus components to the core of the translocation machinery.  Ref: 12177443 Proc Natl Acad Sci U S A, 2002
- **VirB9** , when stabilized by the concurrent expression of  **VirB7** , was shown to be sufficient to permit VirB10 to cross link into its usual high molecular weight forms in the absence of other Vir proteins.  Ref: 8981983 J Bacteriol, 1997
- Strains producing several VirB6.i4 mutant proteins assembled novel  **VirB7**  and  **VirB9**  complexes detectable by nonreducing sodium dodecyl sulfate polyacrylamide gel electrophoresis, and two strains producing the D60.i4 and L191.i4 mutant proteins translocated IncQ plasmid and VirE2 effector protein substrates in the absence of a detectable T pilus.  Ref: 12700266 J Bacteriol, 2003
- Mutational studies demonstrate that cysteine residues at positions 24 of  **VirB7**  and 262 of  **VirB9**  participate in the formation of this complex.  Ref: 8799123 Proc Natl Acad Sci U S A, 1996
- Finally, the membrane distribution and cross linking patterns of VirB10, but not of VirB8 or  **VirB9** , in a strain expressing only  **VirB7**  to VirB10 are significantly altered compared to the patterns of the wild type.  Ref: 12754223 J Bacteriol, 2003
- **VirB9**  and  **VirB7**  are essential components of the putative VirB membrane channel required for transfer of the T complex from Agrobacterium tumefaciens into plants.  Ref: 9023204 J Bacteriol, 1997
- Furthermore, the filamentous structures on bacterium were found to be associated with Hp0532  **VirB7**  or Hp0528  **VirB9** , the major components of TFSS, by immunogold electron microscopy.  Ref: 12780777 Cell Microbiol< MedlineTA>Cell Microbiol, 1997
- Our findings support a model that VirB6 mediates formation of  **VirB7**  and  **VirB9**  complexes required for biogenesis of the T pilus and the secretion channel.  Ref: 12700266 J Bacteriol, 2003
- In the absence of VirB6, cell bound  **VirB7**  monomers and  **VirB7**   **VirB9**  heterodimers were reduced and  **VirB7**  homodimer formation was abolished.  Ref: 10913084 J Bacteriol, 2000
- in turn, the  **VirB7**   **VirB9**  heterodimer promotes the assembly of a functional T complex transport machinery.  Ref: 8655495 J Bacteriol, 1996
- and iii a delta virB operon mutant expressing  **virB7**  from IPTG inducible Pklac and  **virB9**  from an AS inducible PvirB.  Ref: 8655495 J Bacteriol, 1996
- Several virB genes required additional upstream or downstream sequences for complementation i virB2 complemented the delta virB2 mutation only when the complementing plasmid coexpressed virB1 and virB2, ii virB6 and  **virB9**  complemented the delta virB6 and delta  **virB9**  mutations only when the complementing plasmids carried at most 55 and 230 bp of sequences residing 5 of these genes, respectively, and iii  **virB7**  and virB8 complemented the delta  **virB7**  and delta virB8 mutations only when the complementing plasmid coexpressed  **virB7**  and virB8.  Ref: 8206843 J Bacteriol, 1994
- Here, we analyzed the function of 12 cag PAI genes from both cag I and cag II loci, including the complete virB D complex virB4,  **virB7** , virB8,  **virB9** , virB10, virB11, and virD4.  Ref: 11796597 Infect Immun, 2002
- This study characterized the contribution of Agrobacterium tumefaciens VirB6, a polytopic inner membrane protein, to the formation of outer membrane  **VirB7**  lipoprotein and  **VirB9**  protein multimers required for type IV secretion.  Ref: 12700266 J Bacteriol, 2003
- Agrobacterium tumefaciens VirB6 protein participates in formation of  **VirB7**  and  **VirB9**  complexes required for type IV secretion.  Ref: 12700266 J Bacteriol, 2003
- Two presumptive VirB protein complexes, composed of VirB6,  **VirB7** , and  **VirB9**  and of  **VirB7** ,  **VirB9** , and VirB10, were isolated by immunoprecipitation or glutathione S transferase pulldown assays from detergent solubilized membrane extracts of wild type A348 and a strain producing only VirB6 through VirB10 among the VirB proteins.  Ref: 12700266 J Bacteriol, 2003
